# Editorial: Psychological health in the digital age across global communities

**DOI:** 10.3389/fpsyt.2026.1903585

**Published:** 2026-06-22

**Authors:** Xieyining Huang, Tali Gazit

**Affiliations:** 1 Independent Researcher, Atlanta, GA, United States; 2 Bar-Ilan University, Ramat Gan, Israel

**Keywords:** AI Safety, global majority, psychological health, Trust and Safety, user wellbeing

## Why a global majority lens is urgent now

Advancements in Artificial Intelligence (AI) and digital products have largely been driven by a handful of high-income regions (Silicon Valley, Europe, etc.), yet the majority of users live in the Global Majority (emerging and developing economies) (1). Simultaneously, digital adoption is rising fastest in developing markets (2). This imbalance embeds Western cultural assumptions into products that are adopted at scale in the Global Majority, producing unintended but systematic harms, particularly as AI systems are rapidly adopted in diverse contexts where patterns of trust, interpretation, and use may differ meaningfully (3).

Practitioners of Trust & AI Safety, an emerging field that encompasses both traditional Trust & Safety functions (e.g., content moderation) and AI safety efforts aimed at mitigating risks from AI systems, are often globally distributed. Even though many frontline roles (e.g., commercial content moderation positions) are based in the Global Majority, strategy, product, and policy decision-makers remain overrepresented in high-resource countries. This power asymmetry compounds inequity and leaves low-resourced communities particularly vulnerable.

Several Trust & AI Safety leaders have begun to address this gap. For example, the Trust and Safety Foundation hosts a Global Majority Research Committee that collaborates on topics meaningful for the Global Majority (4), and the Journal of Online Trust & Safety recently called for papers to address the unique safety challenges of the Global Majority (1).

While these movements are important, we believe psychologists and psychiatrists also have a unique role in shaping user safety and wellbeing. Our understanding of human behavior can help anticipate product risks, our research on causal mechanisms can inform interventions to mitigate risks, and our science on culture, groups, and communities can shape a more equitable experience. As such, we issued this call for papers with the explicit goal of uplifting Global Majority-led research on psychological health in digital contexts.

## Key themes across the Research Topic

After approximately 18 months of review, we selected 11 papers authored by researchers from 10 nations. These studies examined diverse platforms (Facebook, YouTube, TikTok, Little Red Book/Xiaohongshu, Douyin, WeChat, Weibo, etc.) as well as general internet and social media use. Three broad themes emerged:

Together, this Research Topic shows continuity and novelty. The first two groups reinforce known effects in under-studied cultures. The third cluster highlights continued efforts in understanding manipulated content on a locally relevant topic and the effects of unsafe content on a workforce primarily based out of under-resourced countries.

### Social media engagement and personal wellbeing

These papers largely confirmed familiar findings in local contexts. Social media can be a source of emotional support and community (Isser and Gazit), especially in times of crisis (Steinfeld et al.). Simultaneously, certain design affordances may amplify risks (e.g., infinite scroll: Yue et al.; beauty filters: Sun and Wu).

### Child and youth wellbeing

Reflecting public interest in how technology may place younger users at disproportionate risk, a second cluster of papers focused on the experience of young people. Again, these papers largely confirmed familiar patterns in local contexts. For instance, stronger self-regulation has been found to protect against excessive digital media use among adolescents (Hrbackova et al.), whereas loneliness can both contribute to and result from problematic use (Bai et al.; Ran et al.).

### Safety and wellbeing within content moderation

A third group of papers focused on unsafe content detection, moderation, and what it means for the safety and wellbeing of both platform users and Trust & AI Safety professionals. Analyzing global user-generated reviews on a video game popularized in the Global Majority, Tang et al. found that certain high-engagement content showed a high likelihood of manipulation and coordination. Another pair of papers directly addressed challenges faced by frontline content moderators based in the Global Majority. Savio et al. and de Villa et al. explored the psychological impact on moderators and examined interventions to protect their wellbeing.

## Directions for future research and collaboration

This call for papers represents an important first step in advancing knowledge in this field. However, through the process of reviewing submissions, several critical gaps have become apparent. We therefore urge the field to deepen and broaden this work in several ways.

### Cross-cultural comparative studies

Country-specific studies deserve merit and are important to validate global assumptions related to product and policy that have significant implications for user wellbeing. All the papers included in this Research Topic either selected their samples within specific countries in the Global Majority or included a global pool of participants with substantial representation from the Global Majority. We did not receive any submissions on this topic that directly compared lingual, cultural, and regulatory contexts across locations. Although certain inferences can be made by referencing country-specific studies with similar study designs, it is critical to directly study cross-cultural similarities and differences using designs that can disentangle universal mechanisms from culturally specific effects.

### Cross-border and cross-sector partnerships

Three out of the 11 papers included in this Research Topic involved research collaboration across borders, and one involved collaboration across industry and academia. We truly believe that research can be made better and its impact made stronger when researchers with diverse perspectives come together. Such collaborations come with unique challenges to balance the needs, constraints, and incentives of multiple stakeholders, but successful collaborations can result in profound benefits across settings (5, 6).

## Conclusion: a call to action

The stakes are high. AI and social media are now deeply global technologies, shaping the daily experiences and wellbeing of billions. Yet the products and systems that govern these experiences are often built without sufficient input from those trained to understand human behavior (7). Psychologists and psychiatrists are uniquely positioned to bridge this gap. We cannot remain on the sidelines while psychologically consequential design decisions are made without sufficient behavioral insight. Instead, we must actively engage by conducting research that is both culturally grounded and directly translatable into product design and policy decisions, evaluating systems across diverse cultural contexts, and shaping the policies and practices that define safe and equitable digital environments.
